# Relationship between Diabetes Family Conflicts or Problem Recognition in Illness Self-Management and Quality of Life of Adolescents with T1DM and Their Parents

**DOI:** 10.3390/ijerph182010710

**Published:** 2021-10-13

**Authors:** Mi-Kyoung Cho, Mi Young Kim

**Affiliations:** 1 Department of Nursing Science, Chungbuk National University, Cheongju 28644, Korea; ciamkcho@gmail.com; 2 College of Nursing, Hanyang University, Seoul 04763, Korea

**Keywords:** type 1 diabetes, parent, adolescents, quality of life, diabetes family conflict, PRISM

## Abstract

This study aimed to investigate the relationship between diabetes family conflicts or problem recognition in illness self-management (PRISM) and the parental perceived quality of life (QoL) of adolescents with type 1 diabetes mellitus (T1DM) and their parents. This was a cross-sectional study, and the participants comprised 111 parents of type 1 diabetes adolescents; data were collected via an online survey and analyzed by descriptive statistics, correlation, and multiple linear regression analysis using the IBM SPSS 25.0 program. The explanatory power of the QoL model in parents of adolescents with T1DM, constructed using three variables—diabetes family conflict (B = −0.56), regimen pain and bother (B = −11.25), and peer interactions (B = −7.48), which are PRISM barriers—was 35.7% (F = 5.70, *p* < 0.001). Diabetes family conflicts (B = −0.86) and peer interactions (B = −9.04) explained 57.3% of the variance in the parental perceived QoL of adolescents with T1DM (F = 12.33, *p* < 0.001). In order to improve the QoL in parents and adolescents with type 1 diabetes, interventions to effectively manage diabetes family conflicts and improve peer interactions are necessary.

## 1. Introduction

### Research Motivation

Type 1 diabetes mellitus (T1DM) is an autoimmune disease characterized by insulin deficiency resulting from beta-cell impairment [1]. T1DM is reportedly common across Europe and the United States and less common in Asian countries, including Korea [2]. However, the incidence of T1DM among Korean children and adolescents has been increasing, with the number of cases per 100,000 changing from 32.85 in 2007, to 41.03 in 2017 [2]. Thus, T1DM is a rising problem requiring our attention.

T1DM requires continuous adaptation and management. Managing T1DM is a complex and challenging process that involves diet management, exercise, insulin injections, and blood glucose monitoring [3]. Extra considerations must be made for adolescents with T1DM, who tend to become more independent from their parents and undergo rapid biological and hormonal changes. While transitioning into adolescence, children with T1DM often exhibit poor adherence to treatment and deteriorating metabolic control and are at an increased risk for physiological disorders [4]. Thus, adolescents with T1DM are physiologically and psychologically vulnerable to diseases. As primary care providers who can treat and support vulnerable adolescents with T1DM [5], parents play an important role in its management.

However, parents are also negatively affected by their children’s health. The daily self-management of T1DM requires parents’ participation and can psychologically distress both children and their parents [6]. Psychological distress experienced by parents caring for their chronically ill children negatively affects their disease management [7] and health outcomes [8]. As parents experience clinically significant levels of stress upon learning about their child’s diabetes diagnosis [9], it is necessary to pay attention to not only adolescents with T1DM, but also their parents.

The overall impact of a disease on life can be assessed in terms of quality of life (QoL). QoL is a multi-dimensional concept defined by the World Health Organization (WHO) as “an individual’s perceptions of their position in life, in the context of the culture and value systems in which they live, and in relation to their goals, expectations, standards, and concerns” [10]. A systematic review [11] reported that although the generic QoL of children and adolescents with T1DM was not impaired compared to a healthy group, disease-specific QoL problems, such as a negative impact of diabetes on daily functioning and diabetes-related worries, were present among them. Another study reported a reduced QoL in two-thirds of parents whose children had T1DM [12]. Thus, it is necessary to assess the QoL of parents whose children have T1DM and investigate the affecting factors. 

Unlike the family culture in Western countries emphasizing children’s independence and autonomy since early childhood, the family culture in Korea values the interdependence between parents and children and parents’ involvement in children’s lives [13]. Investigating the parental perceived QoL of adolescents with T1DM can provide clues about how parents perceive their children’s lives. Based on a report that the self-perceived QoL of children with T1DM is dissimilar to the QoL perceived by parents [14], understanding the parental perceived QoL of children with T1DM may provide necessary insights. According to previous studies, psychosocial responses, such as depression, anger, and stress due to T1DM, and factors such as self-management, coping, self-efficacy, and family support, affect QoL [15]. When a person has a chronic disease, their psychological condition must be considered; as it is important to accept and adapt to changes in lifestyle due to the disease [16], it is said that parents have a significant influence on such lifestyle changes.

Although family functioning plays an important role in how adolescents with T1DM adapt to diseases [17,18], children with T1DM are more likely to experience family conflicts related to treatment management following their transition to adolescence [19]. Since diabetes management requires management in all areas of life, related family conflicts may occur [20]. Based on a report that family function impaired by family conflicts negatively affects adaptation to diseases [16], it is necessary to investigate the relationship between diabetes family conflicts, which play an important role in disease management, and the QoL of parents and the parental perceived QoL of adolescents with T1DM. Thus, this study aimed to investigate the relationship between diabetes family conflicts and QoL.

Self-management of T1DM is difficult due to the nature of the disease. To maintain optimal glycemic control, children with T1DM and their parents must follow a complex and demanding daily treatment regime involving blood glucose monitoring, administering a correct dose of insulin at the right time, and regulating food intake and physical activity levels [11]. Self-management of diabetes involves a wide range of tasks, including emotional work, technical tasks, and new responsibilities [7]. To examine the difficulties experienced by parents within these domains, this study investigated how the barriers in each self-management domain affect the QoL. Identifying the domains in which parents struggle and how these domains affect their QoL will provide basic data allowing for a more targeted interventional approach. 

This study examined the QoL of parents of adolescents with T1DM, the parental perceived QoL of adolescents with T1DM, diabetes family conflict, and problem recognition in illness self-management (PRISM). It identified the factors affecting the QoL of parents of adolescents with T1DM and the parental perceived QoL of children with T1DM. This study asked the following questions:

1. What is the QoL of parents of adolescents with T1DM, the parental perceived QoL of these adolescents, diabetes family conflict, and PRISM?
2. How does the QoL of adolescents with T1DM and that of their parents differ according to their general characteristics?
3. What are the correlations among the QoL of parents of adolescents with T1DM, the parental perceived QoL of these adolescents, diabetes family conflict, and PRISM?
4. What are the factors affecting the QoL of parents of adolescents with T1DM and the parental perceived QoL of these adolescents?

## 2. Methods

### 2.1. Study Design

This is a descriptive survey study examining QoL, diabetes family conflict, and PRISM in adolescents with T1DM and their parents and identifying the factors affecting the QoL of adolescents with T1DM and their parents. 

### 2.2. Participants

The participants of this study were parents of adolescents aged ≥13 years and ≤18 years with T1DM, who understood the purpose of this study and voluntarily consented to participate. Only one parent per child could participate in the questionnaire survey. People enrolled in an intervention program aimed at improving the QoL of parents of adolescents with T1DM during the data collection period, and those with cognitive or psychiatric disorders, were excluded from the study. The target sample size was determined at 110 using G*power version 3.1.9.6 [21] with the significance level (α) set at 0.05, the effect size (f2) at 0.21 based on a study by Laffel et al. [22], the power (1-β) at 0.85, and the number of variables at 14. Data were collected from the target number of participants. A total of 111 questionnaires were retrieved and used in the final analysis. 

### 2.3. Research Ethics

This study was approved by the Institutional Review Board (no. EU17-46). The online questionnaire survey conducted in this study was harmless to the participants, who voluntarily participated in the survey after being informed about the purpose and methods of this study by the researchers. Questionnaire data were accessible to only the researchers; furthermore, participants were informed that any personally identifiable data would be anonymized, data collected in this study would not be used for purposes other than the purpose of the study, and they could withdraw their participation at any time without experiencing any disadvantages. 

### 2.4. Tools

#### 2.4.1. Participant Characteristics

Parents’ age, gender, occupation, diabetes education, number of children, rank of DM childbirth, and source of DM information; and age, gender, duration of disease, complications, and glycated hemoglobin (HbA1C) recently measured at a hospital for children with T1DM were collected.

#### 2.4.2. QoL of Parents of Children with T1DM

The Pediatric Quality of Life Family Impact Module (PedsQLTM Family Impact Module) developed by Varni et al. [23] to measure the QoL of parents of patients with T1DM was used. The PedsQLTM Family Impact Module consists of 36 items—28 items related to parental functioning (6 items on physical function, 5 on emotional function, 4 on social function, 5 on cognitive function, 3 on communication, and 5 on worries) and 8 items related to family functioning (3 on daily life and 5 on family relationships). Each question is rated on a five-point scale (0 = “Never” and 4 = “Almost always”). The scale was adjusted such that the score for each item ranged from 0 to 100 points (0 = 100, 1 = 75, 2 = 50, 3 = 25, 4 = 0). Total scores and the score for each domain were divided by the number of items to calculate a mean score out of 100. Higher scores for each domain indicated a higher QoL. The tool had a Cronbach’s α of 0.97 in the study by Varni et al. [23] and 0.96 in this study. 

#### 2.4.3. Parental Perceived QoL of Adolescents with T1DM

The Pediatric Quality of Life Diabetes Module (PedsQLTM 3.0 Diabetes Module) developed by Varni et al. [24] to measure the parental perceived QoL of adolescents with T1DM was used in this study. The tool assesses the QoL of adolescents with T1DM based on parents’ perceived frequencies of various problems experienced by their children as a result of T1DM [24]. The questionnaire consists of 28 items—11 items related to diabetic symptoms, 7 on barriers in diabetes treatment, 4 on treatment adherence, 3 on worries about diabetes, and 3 on communication difficulties related to diabetes. Each item is rated on a five-point scale (0 = “Never” and 4 = “Almost always”). The scale was adjusted such that the score for each item ranged from 0 to 100 points (0 = 100, 1 = 75, 2 = 50, 3 = 25, 4 = 0). The tool had a Cronbach’s α of 0.77 at the time of its development by Varni et al. [24] and 0.90 in this study. 

#### 2.4.4. Diabetes Family Conflict

The Diabetes Family Conflict Scale (DFCS) developed by Rubin et al. [25] and modified by Hood et al. [20] was used to assess diabetes family conflicts in this study. The DFCS measures family conflicts related to diabetes management, including insulin administration, blood glucose measurement, and telling others about diabetes, that are experienced by parents of patients with T1DM. The tool consists of 19 items. Each item is rated on a three-point scale (1 = “never,” 2 = “sometimes,” and 3 = “always”). Total scores range from 19 to 57 points. Higher scores indicate greater family conflicts related to T1DM management. The tool had a Cronbach’s α of 0.85 in a study by Hood et al. [20] and 0.93 in this study. 

#### 2.4.5. Problem Recognition in Illness Self-Management 

The Problem Recognition in Illness Self-management (PRISM) was developed by Cox et al. [26] to identify the barriers experienced by children with T1DM and their parents in diabetes self-management. The tool consists of 25 items across six domains—6 items for understanding and organizing care, 4 for regimen pain and bother, 4 for denial of disease and consequences, 4 for healthcare team interactions, 5 for family interactions, and 2 for peer interactions. This study used the version of the tool developed for parents of adolescents with T1DM. PRISM is a validated tool, and reliability was not presented in the original tool. The Korean version’s validation of the PRISM tool was translated and reverse-translated for bilingual people. Content validity was checked with a nursing professor, an endocrinologist, an outpatient diabetes education nurse, and a nurse with more than 5 years of experience in the diabetes ward. It was checked whether there was any difficulty in answering the questions of two parents with T1DM diabetic children. Each item is rated on a five-point Likert scale (1 = “Not true at all” and 5 = “Very true”). Negatively worded items were reverse-scored. Higher total scores and higher scores for each domain indicate greater self-management barriers experienced by parents of adolescents with T1DM. The sum of the scores for all items in each domain was divided by the number of items to calculate the mean score for each domain. A mean score of ≥2 indicates the presence of barriers to self-management of diabetes in the corresponding domain. A higher mean score indicates that the corresponding domain has a greater impact on the regulation of blood glucose levels in patients with T1DM. In this study, the tool had a Cronbach’s α of 0.90. 

### 2.5. Data Collection

Data were collected through an online survey from 22 April to 3 May 2019. A participant recruitment advertisement and a URL to the online survey were posted on an online T1DM community website after informing the administrators of the community website of the purpose and content of this study and the survey method to gain their consent. Participants were instructed to read the purpose and methods of this study, participants’ rights, and confidentiality before starting the survey. Only those who voluntarily consented to participate were allowed to proceed with the survey. The survey collected data about general characteristics, children-related characteristics, QoL, diabetes family conflict, and PRISM. Participants who completed the survey were rewarded with 10,000 won.

### 2.6. Statistical Analysis

Collected data were analyzed using the IBM SPSS 25.0 program (IBM, Armonk, NY, USA). The characteristics of adolescents with T1DM and their parents were expressed as mean, standard deviation, frequency, percentage, and range. The QoL, diabetes family conflict, and PRISM of adolescents with T1DM and their parents were expressed as mean, standard deviation, and range. The differences in the self-perceived and parental perceived QoL of adolescents with T1DM were analyzed using an independent *t*-test and one-way ANOVA with the Scheffe test as a post-hoc test. The differences in the QoL of adolescents with T1DM and their parents according to the barriers to PRISM were analyzed using an independent *t*-test. For correlation analysis between variables, a normality test was performed using the Shapiro–Wilk test. The correlations among the QoL of adolescents with T1DM and their parents, diabetes family conflict, and PRISM were analyzed using Pearson’s correlation coefficients. The effects of the characteristics of adolescents with T1DM and their parents, diabetes family conflicts, and PRISM on the QoL of adolescents with T1DM and their parents, were examined via simultaneous multiple linear regression analysis. To confirm whether the assumption of multiple linear regression is satisfied, the residual plot was checked for unbiased and homoscedastic linearity. The normality of the residual was confirmed by the normal probability plot, the assumption of equal variance of the residual was confirmed by the Shapiro–Wilk test, and the independence of the error term was confirmed by the Durbin–Watson statistic. The statistical significance level was set at *p* < 0.05. 

## 3. Results

### 3.1. Characteristics of Adolescents with T1DM and Their Parents

The mean age of the parents of adolescents with T1DM was 45.23 ± 3.71 years (range: 38~54 years). Overall, 88 participants were mothers (79.3%), and 70 (63.1%) were employed. Furthermore, 104 parents (93.7%) received education on T1DM management, and 66 (59.5%) had two children. Seventy-one parents (64.0%) had a child with T1DM as their first child, and 94 parents (84.7%) obtained information about T1DM from the Internet. The mean age of the adolescents with T1DM was 15.70 ± 1.76 years (range: 13–18 years), 56 adolescents (50.5%) were male, and the mean duration of disease was 5.76 ± 4.15 years (range: 0–16 years). In total, 62 adolescents (55.9%) had more than 5 years of disease duration and 4 (3.6%) had diabetic complications. The mean HbA1C level was 7.52 ± 1.59% (range: 5.4~15.0%), and 24 adolescents (21.6%) had HbA1C levels ≤6.5% (Table 1).

### 3.2. The QoL of Parents, Parental Perceived QoL of Adolescents with T1DM, DFCS, and PRISM 

The mean total score on the QoL of parents of adolescents with T1DM was 57.01 ± 16.42 (range: 13.89–92.36). The mean score on parent functioning was 56.49 ± 16.95 (range: 15.18–95.54), and that on family functioning was 58.84 ± 17.94 (range: 9.38–100.00). The mean total score on the QoL of parental perceived QoL of adolescents with T1DM was 56.27 ± 12.79 (range: 19.64–83.04). The mean score on diabetes family conflicts was 30.39 ± 7.05 (range: 19.00–55.00; Table 2). 

The total and mean scores on PRISM were 64.16 ± 14.31 (range: 33.00–93.00) and 2.57 ± 0.57, respectively. The mean score exceeded two, indicating that the parents of adolescents with T1DM perceived high barriers to diabetes self-management. Peer interactions were the biggest perceived barrier with a mean score of 3.26 ± 1.04, followed by denial of disease and consequences with a mean score of 3.06 ± 0.63. 

### 3.3. Differences in QoL of Parents of Adolescents with T1DM and Parental Perceived QoL of Adolescents with T1DM According to General Characteristics 

Parents of adolescents with T1DM educated on T1DM management had a higher QoL than those who were not (t = −2.62, *p* = 0.010). Parents with 2–3 children had a higher QoL than those with one child (F = 3.11, *p* = 0.048). Parents whose child with T1DM was the second or third child had a higher QoL than those whose child with T1DM was their first child (F = 3.59, *p* = 0.031). Parents who obtained information about T1DM from the Internet or other sources (books or acquaintances) had a higher QoL than those who obtained the information from hospitals (F = 4.73, *p* = 0.011). No differences in the QoL parents of adolescents with T1DM were found based on other general characteristics (Table 1).

The parental perceived QoL of adolescents with TLDM was higher if parents received education on T1DM management than if they did not (t = −4.05, *p* < 0.001). The parental perceived QoL of adolescents with TLDM was higher if parents obtained information about T1DM management from the Internet or other sources (books and acquaintances) rather than hospitals (F = 17.74, *p* < 0.001), if their child with T1DM had no complications (t = 2.36, *p* = 0.020), and if their child with T1DM had HbA1C levels below the mean HbA1C level of 7.52% (t = 3.20, *p* = 0.002). No differences in the QoL of adolescents with T1DM were found based on other general characteristics (Table 1).

### 3.4. Differences in the QoL of Parents of Adolescents with T1DM and Parental Perceived QoL of Adolescents with T1DM According to Perceived Barriers to Diabetes Self-Management 

Parents of adolescents who responded that they did not perceive understanding and organizing care (t = 4.78, *p* < 0.001), regimen pain and bother (t = 5.50, *p* < 0.001), healthcare team interactions (t = 3.91, *p* < 0.001), family interactions (t = 3.39, *p* = 0.001), and peer interactions (t = 3.00, *p* = 0.003) as barriers to PRISM, had a higher QoL than those who did.

The parental perceived QoL of adolescents with T1DM was higher if parents responded that they did not perceive understanding and organizing care (t = 6.19, *p* < 0.001), regimen pain and bother (t = 4.22, *p* < 0.001), healthcare team interactions (t = 3.81, *p* < 0.001), family interactions (t = 4.05, *p* < 0.001), and peer interactions (t = 4.88, *p* < 0.001) as barriers to PRISM, than if they did (Table 3). 

### 3.5. Correlations among the QoL of Adolescents with T1DM and Their Parents, Diabetes Family Conflicts, and PRISM 

The QoL of adolescents with T1DM was strongly positively correlated with the QoL of parents (r = 0.74, *p* < 0.001). The QoL of parents was negatively correlated with diabetes family conflict (r = −0.48, *p* < 0.001) and PRISM (r = −0.66, *p* < 0.001). The parental perceived QoL of adolescents with T1DM was strongly negatively correlated with diabetes family conflicts (r = −0.66, *p* < 0.001) and PRISM (r = −0.76, *p* < 0.001) (Table 4). 

### 3.6. Factors Affecting the QoL of Adolescents with T1DM and Their Parents 

Table 5 presents the results of a multiple linear regression analysis performed to identify the factors affecting the QoL of parents of adolescents with T1DM and the parental perceived QoL of adolescents with T1DM. Categorical variables including parents’ gender, employment status, prior T1DM management education, number of children, birth order, source of information about T1DM management, gender of the adolescent with T1DM, and T1DM complications were entered as dummy variables. The age of adolescents with T1DM and their parents, duration of disease, and HbA1C levels were entered as continuous variables. Diabetes family conflicts and the six perceived barriers to PRISM were converted to dummy variables and entered as independent variables in the multiple linear regression analysis. Variables were selected using a significance probability of 0.05 and removed using a significance probability of 0.10. The tolerance between the independent variables in the model for predicting the QoL of parents of adolescents with T1DM and the parental perceived QoL of adolescents with T1DM was ≥0.1; the variance inflation factor (VIF) was ≤10, indicating no multicollinearity.

Based on the multiple linear regression model predicting the QoL of parents of adolescents with T1DM, the QoL of parents decreased as diabetes family conflicts increased (B = −0.56, t = −2.35, *p* = 0.021). The QoL of parents decreased if the parents responded that they perceived regimen pain and bother (B = −11.25, t = −3.37, *p* = 0.001) and peer interactions as barriers (B = −7.48, t = −2.15, *p* = 0.034). The three variables, diabetes family conflicts, regimen pain and bother, and peer interaction, explained 35.7% of the variance in the QoL of parents of adolescents with T1DM (F = 5.70, *p* < 0.001). 

Based on the multiple linear regression model predicting the parental perceived QoL, the QoL of adolescents with T1DM decreased as diabetes family conflicts increased (B = −0.86, t = −5.69, *p* < 0.001) and decreased if the parents responded that they perceived peer interactions (B = −9.04, t = −4.09, *p* < 0.001) as barriers. The three variables—diabetes family conflicts, understanding and organizing care, and peer interactions—explained 57.3% of the variance in the QoL of adolescents with T1DM (F = 12.33, *p* < 0.001; Table 5).

## 4. Discussion

This study investigated QoL, diabetes family conflicts, and PRISM among adolescents with T1DM and their parents, the factors affecting the parents’ QoL, and the parental perceived QoL of adolescents with T1DM. 

In this study, the QoL of parents of adolescents with T1DM was positively correlated with the parental perceived QoL of adolescents with T1DM. This result was consistent with a previous study reporting a positive correlation between the two variables [14]. In the analysis of the differences in the QoL of parents of adolescents with T1DM based on general characteristics, parents who did not receive T1DM management education, who only had one child, or whose first child had T1DM, had a significantly lower QoL, demonstrating the importance of T1DM education. These results are consistent with a previous report that mothers whose only child or first child has a chronic disease show reduced post-traumatic growth [27]. Raising their first child is a challenge for mothers. Primipara mothers experience difficulties playing the role of a mother due to their lack of knowledge or experience with raising a child [28]. These difficulties increase if their children are sick. Parents describe having a sick child as “losing the hoped-for-typical healthy child” [9,29]. They may experience a greater sense of loss if their only child or first child is sick. 

In this study, no significant differences in the QoL of parents of adolescents with T1DM were found based on the duration of disease, complications, and HbA1C levels. This result supports previous reports that there were no differences in the post-traumatic growth of parents with chronically ill children according to their children’s diagnosis or disease management [27] and that the post-traumatic growth of parents is not associated with the prior recurrence or current health conditions of children with pediatric cancer [30]. In this study, parents whose children had complications or high HbA1C levels perceived the QoL of their children to be low. These results suggest that parents perceive no differences in their QoL according to their children’s disease or conditions, although they perceive differences in their children’s QoL according to these factors. 

The factors affecting the QoL of parents of adolescents with T1DM, were diabetes family conflicts, regimen pain and bother, and peer interactions in PRISM. The factors affecting the parental perceived QoL of adolescents with T1DM, were diabetes family conflicts, understanding and organizing care, and peer interactions in PRISM. 

The results regarding the factors affecting the QoL of adolescents with T1DM and that of their parents were as follows. First, diabetes family conflicts affected both the QoL of parents and the parental perceived QoL of adolescents with T1DM. This result is consistent with a previous report that diabetes-specific family conflicts affect QoL more significantly than the intensity of treatment [22]. It is also consistent with a report that diabetes-specific family conflicts are highly correlated with poorer metabolic control and more psychological distress (i.e., greater symptoms of parental anxiety and adolescent depression) in diabetic children [31]. Families with diabetic members are vulnerable to family conflicts frequently caused by the chronic stress from T1DM management [32]. The results of this study, showing that family conflicts reduce the QoL of diabetic children and their parents, demonstrate the importance of examining and managing diabetes-related family conflicts. A family-oriented approach to diabetes management is needed because a diabetes diagnosis can impact patients’ families in all aspects of their lives [29]. 

In this study, peer interactions were a barrier to PRISM that affected the QoL of parents of adolescents with T1DM and the parental perceived QoL of these adolescents. Support from peers who understand one’s disease is important, as peer interactions encompass the importance of peers and beliefs about how peers react to one’s illness [24]. The results of this study demonstrate the importance of peer interactions and support a previous finding, that parents whose children have overcome their chronic illness or those who have observed other children overcome the same disease, reported that their children showed higher post-traumatic growth [27]. The results are also consistent with a report that parents’ psychological distress is not only caused by a child’s T1DM diagnosis, but is also affected by the availability of ongoing emotional support [33]. This may be associated with how parents who obtained diabetes-related information from sources other than hospitals had a higher QoL in this study. Hospitals are a mere source of diabetes-related information for parents; however, they may have the opportunity to engage in peer interactions or meet their potential role models when obtaining diabetes-related information from other sources. The positive effect of peer interactions on the QoL of parents observed in this study demonstrates that learning about others’ stories of overcoming T1DM has a positive impact on parents, and that a peer support program or a self-help group involving those who have overcome the same difficulties may improve parents’ QoL.

In this study, parental perceived peer interactions of children were the factors affecting the parental perceived QoL of adolescents. In other words, parents thought that the better their children’s peer interactions, the higher their QoL. This result supports a previous study demonstrating the importance of peer relationships in the psychosocial adaptation of adolescents with T1DM [34]. Adolescents often face high barriers to peer interactions, which are important during adolescence. Children with T1DM experience difficulties due to the lack of awareness about diabetes among teachers and peers [35]. This finding is supported by a report that adolescents with T1DM avoid treatment for the fear of others viewing them as different [36]. These adolescents suffer from the social stigma around diabetic patients. Feeling concerned about the negative view that the public may have toward needles and self-conscious about their behaviors, adolescents with T1DM resort to taking different actions than what they intended when they require injecting their medication in a public place [35]. In the absence of sufficient peer interactions, adolescents with T1DM may experience difficulties in properly managing their disease and suffer from a poor QoL. As the QoL of these adolescents is associated with public assistance [37], a peer support program such as a camp or seminar that would help them receive peer support, or a peer relationship support program such as self-help groups or school interventions, may be useful. 

Regimen pain and bother was a barrier to PRISM affecting only the QoL of parents of adolescents with T1DM. This variable includes feelings about the positive and negative aspects of a self-management regimen [26]. This indicates that one’s QoL is reduced by negative feelings more significantly than by the disease itself or its management. This is consistent with a report that parents of patients with T1DM feel burdened by the prospect of changing their life patterns and participating in daily diabetes management, which involves diet management, blood glucose measurement, and performing insulin injections for their children, who often fear insulin injections and blood glucose checks [38]. This is also consistent with the finding that this emotional burden negatively affects parents’ QoL [39]. These reports suggest that T1DM and its management can negatively affect the QoL of parents. 

Understanding and organizing care was a barrier to PRISM that only affected the parental perceived QoL of adolescents with T1DM. This variable includes knowledge and skills about diabetes and one’s self-management regimen [26]. Parents perceiving understanding and organizing care as a barrier, believed that diabetes self-management affected the QoL of their children. The differences in the parental perceived QoL of adolescents with T1DM observed according to complications and HbA1C levels, suggest that parents tend to focus on the impact of the disease when assessing their children’s QoL.

The parents included in this study perceived barriers in all domains of diabetes self-management, suggesting that they experience difficulties in managing T1DM, especially as its daily management involves a wide range of tasks including frequent insulin injections, blood glucose monitoring, and regulating dietary intake and physical activities [9]. Caregivers of children with diabetes suffer from constant stress and chronic sorrow that does not resolve [40]. Assisting them in diabetes management will not only be of practical help but also provide psychosocial support. As stress from diabetes self-management negatively affects diabetes outcomes, including QoL, self-management, and glycemic control [8,41], diabetes management interventions may have a positive impact on both the physical and psychological well-being of caregivers. Diabetes self-management is a challenging task in which one must manage all aspects of their life. It involves insulin injections, blood glucose monitoring, maintaining a regular diet, and gradually learning how to make decisions regarding exercise and disease management from parents [42].

This study investigated the QoL of parents of adolescents with T1DM and their parental perceived QoL. These adolescents are at an important life stage during which they learn to manage diabetes for the rest of their lives [43]. Children experience great difficulties in forming a sense of identity and independence as they transition into adolescence, during which they undergo various changes and learn to adapt to risks [44]. This study examined the areas of difficulties children face during diabetes self-management. Furthermore, this study is meaningful in that it investigated the parental perceived QoL of children in Korea, which has a strong family-oriented culture emphasizing parent–child bonds. An intervention that can improve peer interactions, reported by parents as a high barrier to diabetes management for both their children and themselves, is needed.

The limitations of this study and suggestions for future research are as follows. 

First, various factors affecting QoL other than the ones measured in this study must be examined. Second, as this study assessed the parental perceived QoL of adolescents with T1DM, future studies can compare the self-perceived and parental perceived QoL of adolescents with T1DM. Third, future studies can examine patients with T1DM from a wider range of age groups. Fourth, this is a cross-sectional study, so caution should be taken in interpreting it as cause and effect. Lastly, a more in-depth longitudinal study on the factors affecting the QoL of adolescents with T1DM and their parents is needed.

## 5. Conclusions

This study examined QoL, diabetes family conflicts, and PRISM in adolescents with T1DM and their parents and identified the factors affecting the QoL of parents and the parental perceived QoL of these adolescents. Diabetes family conflict, regimen pain and bother, and peer interactions explained 35.5% of the variance in the QoL of parents of adolescents with T1DM. Additionally, diabetes family conflicts, understanding and organizing care, and peer interactions explained 55.4% of the variance in the parental perceived QoL of adolescents with T1DM. These results show that a child’s diabetes diagnosis and management significantly affect the child and their family; peer interactions, a barrier to PRISM, affect both the child and their parents. This study is meaningful because it provides basic data that can be used to improve the QoL of adolescents with T1DM and that of their parents. The results demonstrate the need for an intervention aimed at minimizing family conflicts and improving peer interactions. 

## Figures and Tables

**Table 1:** Participant Characteristics and Difference in the Quality of life of Parents and Parental Perceived QoL of Adolescents with T1DM (*n* = 111).

Characteristics	*n* (%) or M ± SD	QoL in Parents		Parental Perceived QoL in Adolescents with T1DM	
		M ± SD	t or F (*p*) *Scheffe* Test	M ± SD	t or F (*p*) *Scheffe* Test
Age of parent (years)	45.23 ± 3.71				
<45	47 (42.3)	55.66 ± 16.80	−0.74 (0.459)	55.03 ± 15.06	−0.87 (0.387)
≥45	64 (57.7)	58.01 ± 16.20		57.17 ± 10.86	
Gender of parent					
Male	23 (20.7)	54.32 ± 18.68	−0.88 (0.379)	53.42 ± 16.43	−0.99 (0.332)
Female	88 (79.3)	57.72 ± 15.82		57.01 ± 11.65	
Occupation of parent					
No	41 (36.9)	57.40 ± 15.28	0.19 (0.850)	57.03 ± 10.89	0.48 (0.630)
Yes	70 (63.1)	56.79 ± 17.16		55.82 ± 13.83	
DM education of parent					
No	7 (6.3)	41.67 ± 25.02	−2.62 (0.010)	38.52 ± 18.81	−4.05 (< 0.001)
Yes	104 (93.7)	58.05 ± 15.31		57.46 ± 11.45	
Number of children					
1 ^a^	32 (28.8)	51.02 ± 18.92	3.11 (0.048)	52.37 ± 16.35	2.14 (0.122)
2 ^b^	66 (59.5)	59.51 ± 15.07	*a < b*	57.95 ± 10.78	
3 ^b^	13 (11.7)	59.08 ± 13.53		57.28 ± 11.06	
Rank of DM childbirth					
1 ^a^	71 (64.0)	53.95 ± 17.20	3.59 (0.031)	55.22 ± 14.07	0.66 (0.521)
2 ^b^	36 (32.4)	62.54 ± 12.63	*a < b*	58.11 ± 9.86	
3 ^b^	4 (3.6)	61.63 ± 22.48		58.26 ± 13.19	
Source of DM information					
Hospital ^a^	15 (13.5)	45.28 ± 19.47	4.73 (0.011)	40.48 ± 14.44	17.74 (<0.001)
Internet ^b^	94 (84.7)	58.87 ± 15.36	*a < b*	58.57 ± 10.49	*a < b < c*
The others ^c^	2 (1.8)	57.99 ± 4.42		66.52 ± 19.57	
Age of child (years)	15.70 ± 1.76				
<15	33 (29.7)	55.41 ± 13.88	−0.67 (0.506)	57.47 ± 10.22	0.64 (0.522)
≥15	78 (70.3)	57.69 ± 17.42		55.76 ± 13.76	
Gender of child					
Male	56 (50.5)	59.67 ± 16.35	1.74 (0.085)	58.50 ± 13.33	1.88 (0.063)
Female	55 (49.5)	54.31 ± 16.19		53.99 ± 11.90	
Duration of disease (year)	5.76 ± 4.15				
<5	49 (44.1)	57.00 ± 14.30	−0.01 (0.995)	56.61 ± 11.83	−0.48 (0.634)
≥5	62 (55.9)	57.02 ± 18.04		56.78 ± 13.57	
Complication					
No	107 (96.4)	57.35 ± 16.50	1.11 (0.270)	56.81 ± 12.56	2.36 (0.020)
Yes	4 (3.6)	48.09 ± 12.48		41.74 ± 11.37	
HbA1C (%)	7.52 ± 1.59				
<6.5	24 (21.6)	61.34 ± 15.12	1.47 (0.145)	60.83 ± 9.05	2.00 (0.048)
≥6.5	87 (78.4)	55.82 ± 16.65		55.01 ± 13.41	

Notes. Superscripts a, b, and c is groups for the *Scheffe* test. TIDM: type 1 diabetes, QoL: quality of life, N: number, M: mean, SD: standard deviation, DM: diabetes mellitus, HbA1C: glycosylated haemoglobin.

**Table 2:** Descriptive Statistics of Variables (*n* = 111).

Variables	Scores		Scale Conversion Scores	
	M ± SD	Min~Max	M ± SD	Min~Max
QoL in Parents	57.01 ± 16.42	13.89~92.36		
Parent functioning	56.49 ± 16.95	15.18~95.54		
Physical functioning	53.15 ± 19.20	12.50~100.00		
Emotional functioning	56.26 ± 21.49	0.00~100.00		
Social functioning	65.20 ± 22.12	6.25~100.00		
Cognitive functioning	64.64 ± 22.47	5.00~100.00		
Communication	58.41 ± 20.24	0.00~100.00		
Worry	44.46 ± 21.58	0.00~100.00		
Family functioning	58.84 ± 17.94	9.38~100.00		
Daily activities	57.06 ± 18.88	8.33~100.00		
Family relationship	59.91 ± 20.87	5.00~100.00		
Parental perceived QoL in adolescents with T1DM	56.27 ± 12.79	19.64~83.04		
Diabetes symptoms	58.48 ± 15.30	22.73~100.00		
Treatment barrier	48.31 ± 11.43	12.50~75.00		
Treatment adherence	54.73 ± 17.23	0.00~100.00		
Worry	61.26 ± 19.93	8.33~100.00		
Communication	57.36 ± 23.59	0.00~100.00		
Diabetes family conflict	30.39 ± 7.05	19.00~55.00	1.60 ± 0.37	1.00~2.90
PRISM	64.16 ± 14.31	33.00~93.00	2.57 ± 0.57	1.32~3.72
Understanding and organizing care	14.01 ± 5.34	6.00~26.00	2.33 ± 0.89	1.00~4.33
Regimen pain and bother	10.96 ± 3.51	4.00~19.00	2.74 ± 0.88	1.00~4.75
Denial of disease and consequences	12.23 ± 2.52	6.00~17.00	3.06 ± 0.63	1.50~4.25
Healthcare team interactions	10.39 ± 3.34	4.00~18.00	2.60 ± 0.83	1.00~4.50
Family interactions	10.06 ± 3.25	5.00~18.00	2.01 ± 0.65	1.00~3.60
Peer interactions	6.51 ± 2.08	2.00~10.00	3.26 ± 1.04	1.00~5.00

Note. TIDM: type 1 diabetes, QoL: Quality of Life, M: mean, SD: standard deviation, PRISM: problem recognition in illness self-management.

**Table 3:** Difference in the QoL of Parents and Parental Perceived QoL of Adolescents with T1DM by PRISM (*n* = 111).

Items of PRISM	N	%	QoL in Parents		Parental Perceived QoL in Adolescents with T1DM	
			M ± SD	t (*p*)	M ± SD	t (*p*)
Understanding and organizing care						
No	49	44.1	64.67 ± 13.89	4.78 (<0.001)	63.39 ± 9.36	6.19 (<0.001)
Yes	62	55.9	50.96 ± 15.82		50.63 ± 12.36	
Regimen pain and bother						
No	27	24.3	70.45 ± 13.41	5.50 (<0.001)	64.68 ± 9.42	4.22 (<0.001)
Yes	84	75.7	52.70 ± 14.95		53.56 ± 12.59	
Denial of disease and consequences						
No	8	7.2	57.99 ± 16.13	0.17 (0.863)	59.04 ± 13.82	0.64 (0.527)
Yes	103	92.8	56.94 ± 16.52		56.05 ± 12.75	
Healthcare team interactions						
No	35	31.5	65.46 ± 14.39	3.91 (<0.001)	62.70 ± 10.64	3.81 (<0.001)
Yes	76	68.5	53.13 ± 15.91		53.30 ± 12.66	
Family interactions						
No	63	56.8	61.42 ± 15.09	3.39 (0.001)	60.29 ± 12.10	4.05 (<0.001)
Yes	48	43.2	51.23 ± 16.45		50.99 ± 11.80	
Peer interactions						
No	20	18.0	66.63 ± 14.72	3.00 (0.003)	67.77 ± 10.84	4.88 (<0.001)
Yes	91	82.0	54.90 ± 16.09		53.74 ± 11.80	

Note. TIDM: type 1 diabetes, QoL: Quality of Life, PRISM: problem recognition in illness self-management, N: number, M: mean, SD: standard deviation.

**Table 4:** Correlations among Variables (*n* = 111).

Variables	QoL in Parents	Parental Perceived QoL in Adolescents with T1DM	Diabetes Family Conflict	PRISM
	*r* (*p*)			
QoL in Parents	1			
Parental perceived QoL in adolescents with T1DM	0.74 (<0.001)	1		
Diabetes family conflict	−0.48 (<0.001)	−0.66 (<0.001)	1	
PRISM	−0.66 (<0.001)	−0.76 (<0.001)	0.71 (<0.001)	1

Note. TIDM: type 1 diabetes, QoL: Quality of Life, PRISM: problem recognition in illness self-management.

**Table 5:** Factors Influencing the QoL of Parents and Parental Perceived QoL of Adolescents with T1DM (*n* = 111).

Model	QoL in Parents			Parental Perceived QoL in Adolescents with T1DM		
	B	SE	t (*p*)	B	SE	t (*p*)
Intercept	78.28	11.20	6.99 (<0.001)	83.02	7.11	11.68 (<0.001)
DM education of parent (ref. = No)						
Yes	3.26	5.89	0.55 (0.581)	5.36	3.74	1.44 (0.154)
Number of children (ref. = 1)						
2	3.84	3.35	1.15 (0.254)	1.59	2.12	0.75 (0.455)
3	1.29	5.77	0.22 (0.824)	1.43	3.66	0.39 (0.697)
Rank of DM childbirth (ref. = 1)						
2	3.87	3.26	1.19 (0.238)	−1.55	2.07	−0.75 (0.455)
3	8.34	8.46	0.99 (0.327)	4.22	5.37	0.78 (0.435)
Source of DM information (ref. = hospital)						
Internet	3.07	9.90	0.31 (0.757)	10.36	6.29	1.65 (0.103)
Diabetes family conflict	−0.56	0.24	−2.35 (0.021)	−0.86	0.15	−5.69(<0.001)
PRISM						
Understanding and organizing care	−2.05	3.35	−.35r (0.541)	−3.72	2.12	−1.75 (0.083)
Regimen pain and bother	−11.25	3.34	−3.37 (0.001)	−01)	2.12	−0.12 (0.091)
Denial of disease and consequences	7.19	5.20	1.38 (0.170)	6.39	3.30	1.94 (0.056)
Healthcare team interactions	−1.54	3.35	−0.46 (0.646)	1.62	2.13	0.76 (0.448)
Family interactions	−2.77	2.93	−0.94 (0.348)	−3.35	1.86	−1.80 (0.075)
Peer interactions	−7.48	3.48	−2.15 (0.034)	−9.04	2.21	−4.09 (<0.001)
Adj. R^2^	0.357			0.573		
F (*p*)	5.70 (<0.001)			12.33 (<0.001)		
Tolerance	0.454–0.900			0.454–0.900		
VIF	1.111–2.205			1.111–2.205		
Durbin-Watson	1.991			1.824		

Note. QoL: Quality of Life, B: unstandardized regression coefficient, SE: standard error, PRISM: problem recognition in illness self-management, VIF: variance inflation factor.

## Data Availability

Data sharing not applicable.

## References

[1] Atkinson M.A., Eisenbarth G.S., Michels A.W. (2014). Type 1 diabetes. Lancet.

[2] Chae H.W., Seo G.H., Song K., Choi H.S., Suh J., Kwon A., Ha S., Kim H.-S. (2020). Incidence and prevalence of type 1 diabetes mellitus among Korean children and adolescents between 2007 and 2017: An epidemiologic study based on a national database. Diabetes Metab. J..

[3] Kumar K.M., Saboo B., Rao P.V., Sarda A., Viswanathan V., Kalra S., Sethi B., Shah N., Srikanta S.S., Jain S.M. (2015). Type 1 diabetes: Awareness, management and challenges: Current scenario in India. Indian J. Endocrinol. Metab..

[4] Hocking M.C., Lochman J.E. (2005). Applying the transactional stress and coping model to sickle cell disorder and insulin-dependent diabetes mellitus: Identifying psychosocial variables related to adjustment and intervention. Clin. Child. Fam. Psychol. Rev..

[5] Jaser S.S. (2010). Psychological problems in adolescents with diabetes. Adolesc. Med. State Art Rev..

[6] Khemakhem R., Dridi Y., Hamza M., Hamouda A.B., Khlayfia Z., Ouerda H., Halioui S., Siala N., Belhadj A., Maherzi A. (2020). How do parents of children with type 1 diabetes mellitus cope and how does this condition affect caregivers’ mental health?. Arch. Pediatr..

[7] Pinquart M. (2018). Parenting stress in caregivers of children with chronic physical condition—A meta-analysis. Stress Health.

[8] Hilliard M.E., De Wit M., Wasserman R.M., Butler A.M., Evans M., Weissberg-Benchell J., Anderson B.J. (2018). Screening and support for emotional burdens of youth with type 1 diabetes: Strategies for diabetes care providers. Pediatr. Diabetes.

[9] Whittemore R., Jaser S., Chao A., Jang M., Grey M. (2012). Psychological experience of parents of children with type 1 diabetes: A systematic mixed-studies review. Diabetes Educ..

[10] WHO QoL Group (1993). Study protocol for the World Health Organization Project to Develop a Quality of Life Assessment Instrument (WHOQOL). Qual. Life Res..

[11] Nieuwesteeg A., Pouwer F., van der Kamp R., van Bakel H., Henk-Jan A., Hartman E. (2012). Quality of life of children with type 1 diabetes: A systematic review. Curr. Diabetes Rev..

[12] Eilander M.M.A., Snoek F.J., Rotteveel J., Aanstoot H.-J., Waarde W.M.B.-V., Houdijk E.C.A.M., Nuboer R., Winterdijk P., De Wit M. (2017). Parental diabetes behaviors and distress are related to glycemic control in youth with type 1 diabetes: Longitudinal data from the DINO study. J. Diabetes Res..

[13] Choi I.J. (2006). Cultural psychological implication of the Korea parent-child relationship. Korea J. Counsel..

[14] Han S.H., Park K.S., Kim K.Y., Ko C.W., Kim H.S., Park Y.H., Kim J.K., Kim H.S., Choi K.H., Hwang J.B. (2013). Reliability and validity of the Korean version of the Pediatric Quality of Life Inventory 3.0 Diabetes Module. J. Korean Soc. Matern Child. Health.

[15] Whittemore R., Jaser S., Guo J., Grey M. (2010). A conceptual model of childhood adaptation to type 1 diabetes. Nurs. Outlook.

[16] Levy M., Burns R.J., Deschênes S.S., Schmitz N. (2017). Does social support moderate the association among major depression, generalized anxiety disorder, and functional disability in adults with diabetes?. Psychosomatics.

[17] Lewin A.B., Heidgerken A.D., Geffken G.R., Williams L.B., Storch E.A., Gelfand K.M., Silverstein J.H. (2006). The relation between family factors and metabolic control: The role of diabetes adherence. J. Pediatr. Psychol..

[18] Whittemore R., Kanner S., Grey M., Melnyk B., Fineat-Overholt E. (2004). The influence of family on physiological and psychosocial health in youth with type 1 diabetes: A systematic review. Evidence-Based Practice in Nursing and Healthcare: A Guide to Best Practice.

[19] Davidson M., Penney E.A., Muller B., Grey M. (2004). Stressors and self-care challenges faced by adolescents living with type 1 diabetes. Appl. Nurs. Res..

[20] Hood K.K., Butler D.A., Anderson B.J., Laffel L.M.B. (2007). Updated and revised Diabetes Family Conflict Scale. Diabetes Care.

[21] Faul F., Erdfelder E., Lang A.-G., Buchner A. (2007). G* Power 3: A flexible statistical power analysis program for the social, behavioral, and biomedical sciences. Behav. Res. Methods.

[22] Laffel L.M., Connell A., Vangness L., Goebel-Fabbri A., Mansfield A., Anderson B.J. (2003). General quality of life in youth with type 1 diabetes: Relationship to patient management and diabetes-specific family conflict. Diabetes Care.

[23] Varni J.W., Sherman S.A., Burwinkle T.M., Dickinson P.E., Dixon P. (2004). The PedsQL™ family impact module: Preliminary reliability and validity. Health Qual. Life Outcomes.

[24] Varni J.W., Burwinkle T.M., Jacobs J.R., Gottschalk M., Kaufman F., Jones K.L. (2003). The PedsQL in type 1 and type 2 diabetes: Reliability and validity of the Pediatric Quality of Life Inventory Generic Core Scales and type 1 Diabetes Module. Diabetes Care.

[25] Rubin R.R., Young-Hyman D., Peyrot M. (1989). Parent-child responsibility and conflict in diabetes care. Diabetes.

[26] Cox E.D., Fritz K.A., Hansen K.W., Brown R.L., Rajamanickam V., Wiles K.E., Fate B.H., Young H.N., Moreno M.A. (2014). Development and validation of PRISM: A survey tool to identify diabetes self-management barriers. Diabetes Res. Clin. Pract..

[27] Kim M.Y., Kim K.-S. (2012). Influence of hope, core beliefs and social support on posttraumatic growth in mothers of chronically ill children. J. Korean Acad. Fundam Nurs..

[28] Kim H.S., Oh K.S., Shin Y.H., Kim T.I., Yoo H.N., Sim M.K., Chung K.H. (2005). Factors influencing parenting stress in primiparas. Korean J. Child. Health Nurs..

[29] Pierce J.S., Aroian K., Caldwell C., Ross J.L., Lee J.M., Schifano E., Novotny R., Tamayo A., Wysocki T. (2017). The ups and downs of parenting young children with type 1 diabetes: A crowdsourcing study. J. Pediatr. Psychol..

[30] Song J.Y., Lee H.K. (2010). The mediating effect of life meaning of childhood cancer patients’ mothers in the link between their hope and posttraumatic growth. Korean J. Counsel..

[31] Williams L.B., Laffel L.M.B., Hood K.K. (2009). Diabetes-specific family conflict and psychological distress in paediatric Type 1 diabetes. Diabetes Med..

[32] Anderson B.J., Vangsness L., Connell A., Butler D., Goebel-Fabbri A., Laffel L.M.B. (2002). Family conflict, adherence, and glycaemic control in youth with short duration Type 1 diabetes. Diabetes Med..

[33] Bowes S., Lowes L., Warner J., Gregory J.W. (2009). Chronic sorrow in parents of children with type 1 diabetes. J. Adv. Nurs..

[34] An S.M. (2016). A Study on Psychosocial Adjustment in Children and Adolescents with Type 1 Diabetes (Master).

[35] Park S.H., Kang H.S., Hwang S.Y., Hwang S.H., Shin Y., Lee J.E. (2012). Insulin self-injection in school by children with type 1 diabetes mellitus. Ann. Pediatr. Endocrinol. Metab..

[36] Richardson A., Adner N., Nordstr¨om G. (2001). Persons with insulin-dependent diabetes mellitus: Acceptance and coping ability. J. Adv. Nurs..

[37] Carlton J., Elliott J., Rowen D., Stevens K., Basarir H., Meadows K., Brazier J. (2017). Developing a questionnaire to determine the impact of self-management in diabetes: Giving people with diabetes a voice. Health Qual. Life Outcomes.

[38] Sahithya B.R., Raman V. (2019). Psychosocial issues in type 1 diabetes mellitus: A review and proposal of a model for evaluation and management in the Indian context. J. Indian Assoc. Child. Adolesc. Ment. Health.

[39] Keklik D., Bayat M., Başdaş Ö. (2020). Care burden and quality of life in mothers of children with type 1 diabetes mellitus. Int. J. Diabetes Dev. Ctries.

[40] Lowes L., Lyne P. (2000). Chronic sorrow in parents of children with newly diagnosed diabetes: A review of the literature and discussion of the implications for nursing practice. J. Adv. Nurs..

[41] Pierce J.S., Kozikowski C., Lee J.M., Wysocki T. (2017). Type 1 diabetes in very young children: A model of parent and child influences on management and outcomes. Pediatr. Diabetes.

[42] Schilling L.S., Grey M., Knafl K.A. (2002). The concept of self-management of type 1 diabetes in children and adolescents: An evolutionary concept analysis. J. Adv. Nurs..

[43] Garvey K.C., Markowitz J.T., Laffel L.M. (2012). Transition to adult care for youth with type 1 diabetes. Curr. Diab. Rep..

[44] Shin Y.M., Cho S.M. (2012). Emotional and behavioral problems in children with chronic physical illness. Ann. Pediatr. Endocrinol. Metab..

